# Modern Strategies to Assess and Breed Forest Tree Adaptation to Changing Climate

**DOI:** 10.3389/fpls.2020.583323

**Published:** 2020-10-21

**Authors:** Andrés J. Cortés, Manuela Restrepo-Montoya, Larry E. Bedoya-Canas

**Affiliations:** ^1^Corporación Colombiana de Investigación Agropecuaria AGROSAVIA, Rionegro, Colombia; ^2^Departamento de Ciencias Forestales, Facultad de Ciencias Agrarias, Universidad Nacional de Colombia – Sede Medellín, Medellín, Colombia

**Keywords:** genomics of adaptation, genomic prediction, genome-wide association studies, genome-wide selection scans, assisted gene flow, machine learning, big data

## Abstract

Studying the genetics of adaptation to new environments in ecologically and industrially important tree species is currently a major research line in the fields of plant science and genetic improvement for tolerance to abiotic stress. Specifically, exploring the genomic basis of local adaptation is imperative for assessing the conditions under which trees will successfully adapt *in situ* to global climate change. However, this knowledge has scarcely been used in conservation and forest tree improvement because woody perennials face major research limitations such as their outcrossing reproductive systems, long juvenile phase, and huge genome sizes. Therefore, in this review we discuss predictive genomic approaches that promise increasing adaptive selection accuracy and shortening generation intervals. They may also assist the detection of novel allelic variants from tree germplasm, and disclose the genomic potential of adaptation to different environments. For instance, natural populations of tree species invite using tools from the population genomics field to study the signatures of local adaptation. Conventional genetic markers and whole genome sequencing both help identifying genes and markers that diverge between local populations more than expected under neutrality, and that exhibit unique signatures of diversity indicative of “selective sweeps.” Ultimately, these efforts inform the conservation and breeding status capable of pivoting forest health, ecosystem services, and sustainable production. Key long-term perspectives include understanding how trees’ phylogeographic history may affect the adaptive relevant genetic variation available for adaptation to environmental change. Encouraging “big data” approaches (machine learning—ML) capable of comprehensively merging heterogeneous genomic and ecological datasets is becoming imperative, too.

## Introduction

How trees will respond to climate change is a pressing question both in the contexts of natural forests and tree plantations (Kremer et al., 2014; Holliday et al., 2017; Isabel et al., 2020). Forests offer key ecological services, boosting significant resources of biodiversity in terms of species and habitats, while help mitigating the impact of excess air pollutants (Phillips et al., 2019; Pennisi, 2020). Trees also source natural renewable materials (i.e., wood itself, cellulose for the pulp industry, and lignin and hemicelluloses for energy production), likely to increase in the future as sustainable alternatives to fossil fuels (Carlson et al., 2014).

Yet, forest tree species are being threatened by climate change (Sullivan et al., 2020) due to fluctuations in the frequency and intensity of heat, drought, salinity (Naidoo et al., 2019), and the incidence of pathogens and pests (Naidoo et al., 2014; Christie et al., 2015). Hence, now more than ever it is essential to explore changing abiotic (Chakhchar et al., 2017; Alcaide et al., 2019b) and biotic (Meyer et al., 2016) interactions. Rampant phenotypic plasticity (Berlin et al., 2017; Hallingback et al., 2019) to climate gradients is presumed in trees, arguing resilience to variability throughout their long lives. Still, forests adaptability should also be assessed in the light of spatially varying local environmental selective pressures (Savolainen et al., 2013), and trees’ genetic and evolutionary potentials (Howe and Brunner, 2005). Both directly reflect and feedback overall adaptive genetic variation. Hence, understanding the genomic drivers that underpin adaptive trait variation becomes vital for conservation and industrial goals.

**FIGURE 1:**
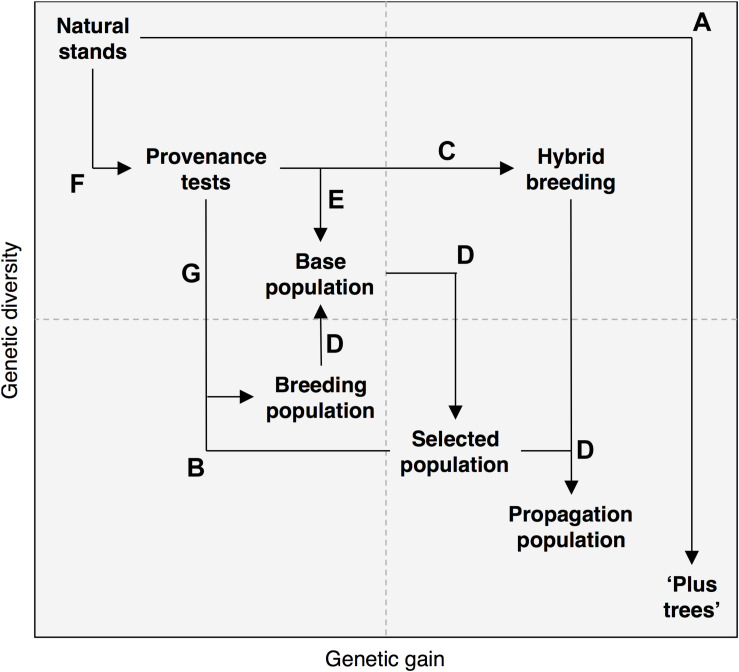
Trans-disciplinary approaches (arrows) such as predictive breeding (GP) and machine learning (ML) promise supporting genome-wide marker-assisted (MAS) pre-breeding and breeding strategies for the selection of **(A)** “plus trees” in the wild, key **(B)** intra- and **(C)** inter- specific parental combinations, and **(D)** elite offspring from those parents. GP and ML should go beyond breeding and feedback **(E)** germplasm utilization and environmental niche classification (Cortés et al., 2013) and enviromics (Costa-Neto et al., 2020; Resende et al., 2020). Genomic-assisted characterizations, such as Genome-Wide Association Studies—GWAS (Neale and Savolainen, 2004), Genome–Environment Associations—GEA (Rellstab et al., 2015; Cortés and Blair, 2018; López-Hernández and Cortés, 2019) and Genome-Wide Selection Scans—GWSS (Zahn and Purnell, 2016), must also start considering more thoroughly **(F)** novel sources of local adaptation, **(G)** genetic-guided infusions and assisted gene flow (AGF), as well an overall systems genetics thinking (Ingvarsson et al., 2016; Myburg et al., 2019).

Developments in plant genomics (Brunner et al., 2007a; Neale and Kremer, 2011) have already disclosed the genetic basis of various useful traits (Khan and Korban, 2012; Tuskan et al., 2018). Yet, this information has limitedly been utilized in tree improvement and conservation (Flanagan et al., 2018), despite genetic gains (Figure 1) and optimized management are urgently required due to environmental issues (Scherer et al., 2020). Besides, breeding woody perennials is primarily bottlenecked by their outcrossing reproductive systems, prolonged juvenile phases (Grattapaglia et al., 2018), large genome sizes lacking elimination mechanisms of long-terminal transposons (Nystedt et al., 2013), and an excessive focus on productivity (Burdon and KlápšTě, 2019) that omits adaptive traits (Table 1; Li et al., 2019). Thus, here we discuss ways to side step these limitations by arguing how predictive genomics can increase selection accuracy and shorten generation intervals (Grattapaglia et al., 2018), assist the detection of exotic variants from tree germplasm (Migicovsky and Myles, 2017), and disclose the genomic potential of adaptation to different climates (Lind et al., 2018). These efforts will ultimately inform conservation and breeding to enhance forest health, ecosystem services, and sustainable production.

**TABLE 1:** Predictive breeding (genomic prediction—GP, also known as genomic selection—GS) studies in forest tree species published during the last years.

**Species**	**Populations**	**Trait data**	**Genotyping data**	**GP algorithm**	**Key conclusions**	**References**
*Elaeis guineensis*	162 individuals from the Deli and Group B populations	Seven oil yield components	262 SSRs	PBLUP, GBLUP	Genomic selection (GBLUP) calibrated according to conditions of the experiment showed higher trait precision when using pedigree-based model	Cros et al., 2015
*Elaeis guineensis*	A × B hybrid progeny tests with almost 500 crosses for training and 200 crosses for independent validation	Seven oil yield components	(>5,000 GBS-derived SNPs	GBLUP, PBLUP	Preselection for yield components using GBS is the first possible application of GS in oil palm.	Cros et al., 2017
*Hevea brasilensis*	332 clones from the F1 cross PB 260 × RRIM 600	Rubber production	332 SSRs on site 1 and 296 SSRs on site 2	RKHS, BLR_A, RR-BLUP-A, BLR_AD, RR-BLUP_AD	Mean between-site GS accuracy reached 0.561 when using the 125–200 SSRs with the highest Ho. The simulations showed that by applying a genomic preselection among 3,000 seedlings in the nursery there is a greater precision of selection of the genomic preselection compared to the phenotypic preselection. Statistical method had no effect on GS precision	Cros et al., 2019
*Eucalyptus grandis* × *E. urophylla* hybrids	999 individuals from 45 families	Cellulose content, composition of lignin monomer, total lignin, WD	33,398 SNP	ABLUP, GBLUP, ssGBLUP	ssGBLUP is a tool with a great projection for the improvement of the precision and the bias of the classic GBLUP for the genomic evaluation in the improvement of *Eucalyptus*	Cappa et al., 2019
*Picea abies*	1,370 controlled-pollinated individuals from 46 unrelated parents	Quality features of solid wood, pilodyn penetration, acoustic speed	116,765 SNP	ABLUP-A, ABLUP-AD, GBLUP-AD, GBLUD-ADE	GBLUP-AD is a model with great utility in production and propagation. Tree breeders can use it for seedling selection, or family and full-siblings selection	Chen et al., 2019
*Eucalyptus globulus*	646 individuals out of approximately 10 individuals per family	WD, branch quality, DBH, HT	14,442 SNP	BRR, Bayes C, HAP, HAP-SNP	In general, the BRR and Bayes C methods had a higher predictive capacity for most of the traits. In particular, genomic models that included the haplotype effect (either HAP or HAP SNP) significantly increased the AP of traits with low heritability.	Ballesta et al., 2019
*Eucalyptus cladocalyx*	1,470 individuals from 49 families	DBH, HT, BHT, WD, STR, SLD, FI	3.8 K Illumina Infinium EUChip60K SNPs	Bayes A, Bayes B, Bayes C, BRR	An GSq approach outperformed GS models in terms of predictive ability when the proportion of the variance explained by the significant marker-trait associations was higher than those explained by the polygenic background and non-significant markers	Ballesta et al., 2020
*Eucalyptus* clones of *E. urophylla*× *E. grandis*	1,130 clones of 69 full- sib families	Biomass production, WUE, wood properties	3,303 SNPs	GBLUP	The inclusion of wood δ13C in the selection process may lead to *Eucalyptus* varieties adapted to marginal zones still presenting good performance for biomass and wood chemical traits	Bouvet et al., 2020
*Picea abies*	726 trees of 40 families of complete siblings from two localities	Density, microfiber angle, wood stiffness	5,660 Infinium iSelect SNP matrix SNPs from exome capture and sequencing	Single-trait: GBLUP, BRR, GBLUP, TGBLUP, ABLUP. Multi-traits: GBLUP	Genomic prediction models showed similar results, but the multi-trait model stood out when weevil attacks were not available. Most of the results indicate that the weevil resistance genotypes were higher when there was a greater proportion of height to diameter and greater rigidity of the wood.	Lenz et al., 2020
*Pinus radiata*	457 POP2 descendants of 63 parents, and 524 POP3 descendants of 24 parents	Branching frequency, stem straightness, internal verification, and external bleeding	1,371,123 exome sequencing capture SNPs	GBLUP, ABLUP	An efficient way to improve non-key traits is through genomic selection with a pedigree corrected using SNP information	Li et al., 2019
*Pseudotsuga menziesii*	13,615 individuals	HT, 13 environmental variables	66,969 SNPs	ssGBLUP	GS-PA can be substantially improved using ECs to explain environmental heterogeneity and G × E effects. The ssGBLUP methodology allows historical genetic trials containing non-genotyped samples to contribute in genomic prediction, and, thus, effectively boosting training population size which is a critical step	Ratcliffe et al., 2019
*Shorea platyclados*	356 individuals from a half-sib progeny population	Seven important traits, including growth, branching quality, wood quality traits	5,900 Illumina Hi-Seq X SNPs	rrBLUP	Selective breeding for these traits individually could be very effective, especially for increasing the diameter growth, branch diameter ratio and wood density simultaneously	Sawitri et al., 2020
*Hevea brasiliensis*	435 individual rubber trees at two sites. 252 F1 hybrids derived from a PR255 × PB217 cross, 146 F1 hybrids derived from a GT1 × RRIM701 cross, 37 genotypes from a GT1 × PB235 cross, and 4 testers (GT1, PB235, RRIM701, and RRIM600)	SC	30,546 GBS-derived SNPs	BLUP, SM, MM, MDs, Mde	Multi-environment models were superior to the single-environment genomic models. Methods in which GS is incorporated resulted in a fivefold increase in response to selection for SC with multi-environment GS (MM, MDe, or MDs)	Souza et al., 2019
*Fraxinus excelsior*	1,250 individuals	Tree health, ash dieback resistance	100–50,000 HiSeq X SNPs	RR-BLUP	Ash dieback resistance in *F. excelsior* is a polygenic trait that should respond well to both natural selection and breeding, which could be accelerated using genomic prediction	Stocks et al., 2019
*Eucalyptus nitens*	691 individuals	Solid wood production, height, DBH, stem straightness, WD, wood stiffness, wood shrinkage, growth strain	12,236 Illumina EUChip60K SNPs	BLUP, GBLUP	The greatest improvement in genetic parameters was obtained for tangential air-dry wood shrinkage and growth strain	Suontama et al., 2019
*Pseudotsuga menziesii*	A 38-year-old progeny test population (P1), selecting 37 of 165 families with complete siblings at random from 3 different settings. Validation population contained 247 descendants with controlled crosses from the 37 families	HT	Complete genotyping of exome capture	RR-BLUP, GRR, Byes-B	The validation of cross genomic selection of juvenile height in Douglas fir gave very similar results with the ABLUP predictive precision, but this precision may be linked to the relationship between training and validation conjugates	Thistlethwaite et al., 2019a
*Pseudotsuga menziesii*, *Picea glauca*, *P. engelmannii*	1,321 Douglas-fir trees, representing 37 full-sib F1 families and 1,126 interior spruce trees, representing 25 open-pollinated (half-sib) families	Mid-rotation height, WD	200–50,000 Illumina HiSeq 2000 SNPs	RR-BLUP	Reducing marker density cannot be recommended for carrying out GS in conifers. Significant LD between markers and putative causal variants was not detected using 50,000 SNPs	Thistlethwaite et al., 2020
*Pinus contorta*	Half- and full- sibs represented by 57 base parents and 42 full-sib families with an calculated effective population size of 92	Growth and wood quality	51,213 Illumina HiSeq SNPs	Bayes C, Bayes B, BLUP, GBLUP, ABLUP	The predictions of Marker-based models had accuracies that were equal to or better than pedigree-based models (ABLUP) when using several cross-validation scenarios and were better at ranking trees within families	Ukrainetz and Mansfield, 2020
*Castanea dentate*	7,173 descendants of BC3F3 from 346 “Clapper” mothers and 198 “Serious” mothers. For the BC3F2 progeny, a total of 1,134 “Clapper” and 1,042 “Graves” were sampled	*Cryphonectria parasitica* fungus severity (BC3F3) or presence/absence data (BC3F2)	Sequencing of a *C. dentata* clone in the PacBio Sequel platform	HBLUP, ABLUP, Bayes C	By means of genomic prediction and estimation of hybrid indices, a trade-off is between resistance and a proportion of inherited genome. The results found show that the genetic architecture underlying the heritability of resistance to blight is complex	Westbrook et al., 2020
*Picea abies*	484 progeny trees from 62 half-sib families	WD, MOE, MFA	130,269 Illumina HiSeq 2500 SNPs	ABLUP, GBLUP, rrBLUP, BayesB, RKHS	This study indicates standing tree-based measurements is a cost-effective alternative method for GS. Selection for density could be conducted at an earlier age than for MFA and MOE	Zhou et al. (2020)

*For a comprehensive summary of previous studies not included here see Grattapaglia et al. (2018). Detailed abbreviations are shown at the end of the table. WUE, water use efficiency; SC, stem circumference; WD, wood density; MOE, modulus of elasticity; MFA, microfibril angle; DBH, diameter at breast height; HT, total tree height; BHT, first bifurcation height; STR, stem straightness; SLD, slenderness index; FI, flowering intensity; SNP, single nucleotide polymorphism; SSR, simple sequence repeat; GBS, genotyping by sequencing.*

## Predictive Breeding Promises Boosting Forest Tree Genetic Improvement

The aim of forest tree breeding is rarely to develop new varieties, but instead advance gradual population improvement through recurrent selection and testing (Neale and Kremer, 2011). Because of the long generation times of forest trees, their breeding has traditionally relied on phenotypic selection from natural stands by choosing “plus-trees” (Figure 1A). Their superior phenotype (primarily productivity and tree architecture, and seldom adaptability) is often measured *in situ* or in provenance trials. This starting pool of preferred trees constitutes the base population, an arboretum from which further selection is carried out to build a selected population with elite seed/scion donors. Their estimated combinatory ability is gathered from genetic tests such as progeny trials, and parental re-selection (Figure 1B) from top families and single trees (White et al., 2007). After three steps of selection (from the natural, base, and selected populations), eroded genetic diversity may jeopardize overall population’s productivity and resilience due to inbreeding depression. In order to minimize this risk, a breeding population is established to increase genetic variability. Intermating may rely on infusions from external populations. Outbred multi-parental populations (Scott et al., 2020) hence become the base population of a second generation. A bottleneck of this approach is that each generation would last at least nine or 18 years, for seedling or elite clone identification, respectively, in a fast growing tree species such as *Eucalyptus* (Resende et al., 2012).

Shortcuts to speed up the traditional cycle of forest tree genetic improvement rely on hybrids and backcrossing. Hybrid breeding (Figure 1C) aims harnessing heterotic effects (hybrid vigor) due to dominance and over-dominance already existing in nature, capable of increasing yield and adaptability (Schilthuizen et al., 2004; Seehausen, 2004). Dominance refers to the masking of deleterious effects of recessive alleles as a consequence of the increased heterozygosity resulting from hybridization (i.e., an scape from inbreeding depression). On the other hand, over-dominance corresponds to the increase in aptitude as the result of the additive and epistatic effects of alleles that are naturally maintained by balancing selection and only coincide in hybrid genotypes. Hybrid breeding is nowadays widely used at operational plantations to maximize circumference at breast height (e.g., *E. grandis*× *E. nitens* and *Pinus elliotti* × *P. oocarpa*), height (e.g., *P. caribaea* × *P. tecunumanii*) and resistance to *Fusarium* spp. (i.e., *P. patula* × *P. tecunumanii*), among other potential uses (Burkhart et al., 2017). Backcrossing helps targeting the introgression of desired traits from exotic sources into elite populations, as has been done to transfer resistance to chestnut blight into American populations from Chinese wild donors (Cipollini et al., 2017).

Molecular breeding approaches (Badenes et al., 2016), in which genetic markers are used to assist selection, offer promising alternatives to speed up traditional tree breeding cycles, as well as hybrid and backcrossing schemes. Marker-Assisted Selection—MAS (Butcher and Southerton, 2007; Muranty et al., 2014) and Backcrossing—MAB (Herzog and Frisch, 2011) provide frameworks to pyramid target genetic variants of simple Mendelian traits, which are those regulated by few major genes (e.g., resistance to biotic stresses). Gene editing (Doudna and Charpentier, 2014; Dort et al., 2020) and transgenics (Campbell et al., 2003) can also transfer or silence allelic variants of major effects within a single generation (Pereira-Lorenzo et al., 2019). These may replicate the success of tolerant chestnuts (Alcaide et al., 2019a; Westbrook et al., 2019) and promote reproductive sterility (Meilan et al., 2001; Fritsche et al., 2018). Yet, molecular breeding via MAS, MAB and gene editing is often inefficient to trace quantitative traits as growth and adaptation to abiotic stresses. Adaptation is often polygenic (Cortés et al., 2018b; Barghi et al., 2020) due to many low-effect genes and their second-order interactions (Boyle et al., 2017).

A last-generation predictive breeding (Figure 1D) approach designed for quantitative polygenic traits is known as Genomic Prediction—GP (Desta and Ortiz, 2014; Crossa et al., 2017; Grattapaglia et al., 2018). GP standardizes infinitesimal marker-based additive predictive models by relying on historical phenotypic data (Meuwissen et al., 2001; Gianola et al., 2006; de los Campos et al., 2013). Trait data must be in Linkage Disequilibrium—LD or genetic auto-correlation (e.g., Kelleher et al., 2012), with the molecular markers or with the samples’ genetic co-ancestry. GP utility has been demonstrated (Table 1) in model forest tree species such as *Eucalyptus* (Resende et al., 2012; Suontama et al., 2019), and conifers as *Pinus* (Resende M. F. et al., 2012; Li et al., 2019) and Douglas-fir (Thistlethwaite et al., 2017, 2019b), but also in non-model perennial crops such as coffee (Sousa et al., 2018), rubber (Cros et al., 2019; Souza et al., 2019) and oil palm (Cros et al., 2015). GP may even fit epigenetics (Roudbar et al., 2020), as well as multi-trait genomic models as was recently confirmed in Norway spruce for growth, wood quality and weevil resistance traits (Lenz et al., 2020). GP could also be coupled with somatic embryo-genesis for clonal propagation of elite genotypes by selecting elite zygotic embryos based on their genomic breeding value (Grattapaglia et al., 2018). GP has the potential to predict untested hybrid genotypes (Technow et al., 2014) in woody perennials (Cros et al., 2017; Tan et al., 2017) by genotyping potential parental lines and phenotyping few F1 hybrids. Prioritizing inter-specific combinations for field trials can speed up hybrid breeding. Meanwhile, like already envision for chestnut (Westbrook et al., 2020), Genomic-Assisted Backcrossing (GABC) will replace MAB as the strategy to assist introgression breeding into elite populations from exotic germplasm.

## Assisting Genomic Characterization of Tree Germplasm to Capture Novel Variants

Exploiting tree wild populations for genomics-assisted breeding (Figure 1E) is key to broaden the genetic basis of woody perennial breeding programs (Migicovsky and Myles, 2017). Specifically, diverse seed bank collections and novel tree provenances might source (Ulian et al., 2020) exotic variation (e.g., unique wood quality properties). They also help avoiding genetic erosion (e.g., via infusions) and increasing long-term adaptability to climate change (e.g., making forests more tolerant to abiotic stresses such as drought and heat). For example, genomic diversity analyses helped capturing rare variants in *P. trichocarpa* germplasm (Piot et al., 2019) often missed by Genome-Wide Association Studies (GWAS) in the related species *P. tremula* (Khan and Korban, 2012). Expanded phylogenomic (Wang M. et al., 2020) and species (Wang et al., 2020) diversity may source novel alleles to support selective breeding, as in wood quality traits for improved bioenergy feedstock. In turn, GP might go beyond breeding, the focus of the previous section, and feedback seed bank characterization (Hickey et al., 2017)—e.g., by predicting seed traits (Kehel et al., 2020) and overall yield (Crossa et al., 2007, 2016) in diverse accessions that otherwise could not have been tested at once in genetic field trials. Although the use of GP for germplasm characterization is latent, it has not been fully explored in forest tree species, a main research gap to be filled in the oncoming years.

Tree species rich in evolutionary diversity (Shang et al., 2020) could leverage breeding. Hybridization (Nieto Feliner et al., 2020), introgression (Burgarella et al., 2019), and polyploidy (Mason and Wendel, 2020) have already pumped morphological novelty by testing more genetic compatibilities than humans ever will. Yet, genomics of adaptive radiations (Seehausen, 2004; Madriñán et al., 2013; Cortés et al., 2018a; Marques et al., 2019) are challenging (Schilthuizen et al., 2004; de la Harpe et al., 2017). Long-living oaks—*Quercus* (Plomion et al., 2018; Leroy et al., 2020b; Plomion and Martin, 2020) are a classical syngameon (Cannon and Petit, 2020) – a promiscuous network of weakly isolated species that has driven peerless historical (Crowl et al., 2020; Hipp et al., 2020; Leroy et al., 2020c) and current (Leroy et al., 2020a) adaptive introgression (Kremer and Hipp, 2020).

In short, marker-assisted schemes are liable to be implemented at various stages during pre-breeding—e.g., in the selection of “plus trees” from the wild (De Dato et al., 2018), of target parental pairs (Blair et al., 2013), and of superior offspring (Galeano et al., 2012). These approaches also aid conservation (Martín et al., 2012; Mattioni et al., 2017) and germplasm tracing (Cortés et al., 2011; Blair et al., 2012; Chiocchini et al., 2016). Still, genomic-assisted studies of germplasm may risk focusing on productive traits and disregard locally adapted trait variation.

## Genomics of Adaptation to Different Environments

Local genetic adaptation (Figure 1F) may prove useful in the reaction of forests to climate change (Savolainen et al., 2013; Lascoux et al., 2016), for instance via gene swamping of pre-adapted alleles (Kremer et al., 2014; de Visser et al., 2018). Nowadays there is a wide portfolio of genomic tools that appeal to environmental variables in order to infer the genetic basis of adaptation to abiotic stresses. Specifically, Genome-Wide Selection Scans—GWSS (Zahn and Purnell, 2016) and Genome–Environment Associations – GEA (Rellstab et al., 2015) aim detecting signatures of selection across environmental gradients by pinpointing sections in the genomes that correlate with habitat heterogeneity (Forester et al., 2016). These approaches have successfully been used to assess variation in bud-break phenology (McKown et al., 2018) and stomata patterning (McKown et al., 2014) as potential responses to climate warming in natural populations of *P. trichocarpa*. They have also allow comparing the likelihoods of adaptive reactions at continental (Holliday et al., 2011; Evans et al., 2014; Zhou et al., 2014; Stölting et al., 2015) and regional scales (Eckert et al., 2010; Holliday et al., 2016; Pluess et al., 2016; Ingvarsson and Bernhardsson, 2020) across phylogenetically diverse taxa (Yeaman et al., 2016). Currently there are even multi-scale approaches to detect widespread divergent selection in non-model tree species experiencing population decline (Mayol et al., 2020).

Local adaptation to climate change can be further enhanced (Figure 1G) via assisted gene flow—AGF (Aitken and Whitlock, 2013). AGF aims minimizing endogenous negative, while maximizing exogenous positive, selection by trans-locating pre-adapted individuals to facilitate adaptation of planted forests to climate change (Aitken and Bemmels, 2016). Management of local adaptation in a changing climate was recently examined in populations from lodgepole pine (*P. contorta*) across western Canada (Mahony et al., 2020). Yet, operational uses of genomic data to guide seed transfer or AGF are still lacking. Alternatively, genetic containment may be desired for transgenic trees (Brunner et al., 2007b; Klocko et al., 2016). The utility of these approaches in tropical forests remains to be explored. Tropical trees are more at risk from warming because they are closer to upper thermal limits (Freeman et al., 2020; Sentinella et al., 2020), as in montane (Cortés and Wheeler, 2018; Feeley et al., 2020; Tito et al., 2020) and alpine (Wheeler et al., 2014, 2016; Valencia et al., 2020) habitats. Disclosing the genetic, pan-genomic (Bayer et al., 2020), and epigenetic (Brautigam et al., 2013; Sow et al., 2018; Barrera-Redondo et al., 2020) bases of traits underlying adaptive responses in tree species will assist AGF, industrial milestones, and conservation priorities (Isabel et al., 2020) across meta-populations (Gonzalez et al., 2020), and even micro-habitats (Cortés et al., 2014; Abdelaziz et al., 2020).

## Concluding Remarks

A major question in the interface between forests and their environments that genomics have the potential to assist is whether tree adaptation to the fast pace of climate change can happen despite their long generation times (Holliday et al., 2017). Specifically, GP offers a feasible way to predict adaptation from allele frequencies in many genes of low effects underlying polygenic traits (Isabel et al., 2020). This way, the role of adaptive responses can be balanced in relation with range shifts (i.e., migration) and extinction as possible climate change outcomes for tree populations (Aitken et al., 2008; Alberto et al., 2013). This question is equally insightful for domesticated and wild stands of forest trees, and must be coupled with reflections regarding the best propagation and conservation schemes. For instance, the factual consequences on genetic diversity of clonal and seedling forestry (Ingvarsson and Dahlberg, 2018), and of assisted gene flow (Aitken and Whitlock, 2013; Aitken and Bemmels, 2016), must be compiled.

Forest genomics tends focusing on economically important species. Yet, the power of population genomics must be further extended to comprehend neutral and adaptive processes in non-commercial species of ecological value in order to advance not just productivity, but also climate adaptation, forest health and conservation (Isabel et al., 2020). In this sense, GP is starting to permeate novel non-key traits other than growth and wood density, but still of interest for breeding, such as branching, stem straightness and external resin bleeding (Li et al., 2019). GP is also predicting adaptive trait variation for abiotic (Eckert et al., 2010) and biotic (Westbrook et al., 2020) stresses. In parallel to an enrichment of target traits, emerging genomic technologies might unlock woody plant trait diversity beyond the model tree species poplar, eucalyptus, willow, oak, chestnut and pecan (Tuskan et al., 2018).

There is currently a rich mosaic of alternative genetic methods to carry out both explicit (direct) and implied (indirect) selection on economic- (Burdon and KlápšTě, 2019) and ecological-worth (Holliday et al., 2017; Isabel et al., 2020) functions. These different traits can enlighten our understanding of the consequences of genetic divergence on the reaction of tree populations to climate change (Kremer et al., 2014). However, novel methodological developments should target more comprehensively complex trait–environment relationships (Bruelheide et al., 2018). They should also mingle between adaptive (Cortés et al., 2015b; Sedlacek et al., 2016) and range shift (Sedlacek et al., 2014; Wheeler et al., 2015) responses across altitudinal (Lenoir et al., 2008; Steinbauer et al., 2018), latitudinal (Chen et al., 2011) and micro-habitat (Sedlacek et al., 2015; Little et al., 2016) gradients.

## Perspectives

Exploring natural adaptation to changing climate and genetic breeding for tolerance to abiotic stress in forest tree species has traditionally been assisted by GWAS, GWSS, GEA (Cortés et al., 2020), and AGF techniques. These approaches have allowed identifying and utilizing naturally available, locally adapted, variants. More recently, major developments in the field of predictive breeding (i.e., GP) promise to speed up selection from natural sources, as well as within the breeding cycle, by shortening the generation intervals and increasing the selection accuracy prior field trials. We have already identified and discussed major improvements in this line, such as multi-trait GP models (Lenz et al., 2020), coupled with integrative selection scores (Burdon and KlápšTě, 2019) on novel non-key (Li et al., 2019) and ecological-worth (Holliday et al., 2017; Isabel et al., 2020) traits. These innovations can capture multi-scale trait–environment relationships (Bruelheide et al., 2018) in non-model tree species (Mayol et al., 2020). Given the complexity and heterogeneity of trans-disciplinary data sources, Machine Learning (ML) offers a timely predictive and synthetizing approach capable of merging the highlights of the GWAS, GWSS, GEA, AGF and GP techniques.

“Supervised” ML typically utilizes “labeled” training datasets in order to cross-validate the “recall” rate of a target classification (e.g., selection). ML powerfully handles high-dimensional inputs of heterogeneous “features” without a joint probability distribution (Schrider and Kern, 2018). This way, algorithmically generated non-parametric models that avoid rejection sampling sidestep the “curse of dimensionality” and offer new ways to reveal complex systems (Myburg et al., 2019). ML has historically been utilized in functional genomics (Libbrecht and Noble, 2015) and ecological niche modeling (Phillips et al., 2017). Yet, it is now transitioning into GWAS-coupled MAS (Cortés et al., 2015a), GP (Crossa et al., 2019; Abdollahiarpanahi et al., 2020), GWSS (Schrider and Kern, 2018), and demographics—as when coupled with Approximate Bayesian Computation (Elleouet and Aitken, 2018; Liu et al., 2019).

We anticipate that ML techniques will brace GP predictions for various traits in multi-environment trials that aim disentangling the additive genetic variance and the genotype × environment components. Novel developments in the field of ML will further allow building more accurate predictions by merging environmental variables, microhabitat diversity, and genome-wide divergence, all within a tree-breeding context to pivot “plus tree” selection, hybrid breeding and GABC schemes, as well as in terms of adaptation to climate change in natural forests. Integrative assessments (Ingvarsson et al., 2016) via ML promise harnessing adaptive trait variation in forest tree species.

## Author Contributions

AC conceived this review. MR-M and LB-C collected literature and prepared summary tables. AC wrote the first draft of the review with later edits made by MR-M and LB-C.

## Conflict of Interest

The authors declare that the research was conducted in the absence of any commercial or financial relationships that could be construed as a potential conflict of interest.
